# Long-term Survival in Hilar Cholangiocarcinoma also Possible in Unresectable Patients

**DOI:** 10.1007/s00268-012-1638-5

**Published:** 2012-05-09

**Authors:** Anthony T. Ruys, Steven van Haelst, Olivier R. Busch, Erik A. Rauws, Dirk J. Gouma, Thomas M. van Gulik

**Affiliations:** 1 Department of Surgery, Academic Medical Center, University of Amsterdam, Meibergdreef 9, 1105 AZ Amsterdam, The Netherlands; 2 Department of Gastroenterology and Hepatology, Academic Medical Centre, Amsterdam, The Netherlands

## Abstract

**Background:**

Radical resection remains the only curative treatment for hilar cholangiocarcinoma (HCCA). Only a limited proportion of patients, however, are eligible for resection. The survival and prognostic factors of these patients are largely unknown. The aim of this study was to evaluate survival and prognostic factors in unresectable patients presenting with HCCA.

**Methods:**

We performed a cohort study of the denominator of HCCA patients seen in a tertiary referral center between March 2003 and March 2009. Demographics, treatment, pathology results, and survival were analyzed.

**Results:**

A total of 217 patients with suspected HCCA were identified. Ninety-five patients (40 %) underwent laparotomy, and in 57 (63 %) of these patients resection was performed. Overall median and 5-year survival of resected patients were 37 months and 43 %, respectively, as compared to 13 months and 7 % in unresectable patients. In unresectable patients, median survival was better in patients with locally advanced disease (16 months) as compared to patients with hepatic and extrahepatic metastases (5 and 3 months, *p* < 0.001). Of the 160 unresectable patients, 17 (10 %) survived longer than 3 years.

**Conclusion:**

Of the patients presenting with HCCA in our center, 26 % proved resectable. The 7 % long-term survival rate of unresectable patients is remarkable and emphasizes the indolent growth of some of these tumors. Patients with metastases had a much worse prognosis with a median of 4 months.

## Introduction

Survival data regarding resected hilar cholangiocarcinoma (HCCA) are well described, with 5-year survival rates varying from 20 to 40 % in most series in recent literature [1, 2]. Many clinical, surgical, and pathological factors have been shown to impact long-term outcome, including negative histological margin status, concomitant hepatic resection, nodal status, well-differentiated tumor grade, papillary tumor morphology, and lack of perineural invasion [2, 3]. However, these studies included only those patients who had undergone resection so, consequently, these study populations are highly selected.

Much less is known about the survival and prognostic factors of unresectable patients, and although survival is mentioned in some papers [4–8], no prognostic factors for unresectable patients have been reported. Indeed, most patients with hilar cholangiocarcinoma are found unresectable at either presentation, after diagnostic laparoscopy, or during exploration [9]. Criteria for unresectable disease include locally advanced tumor, distant metastases, and lymph node metastases beyond the hepatoduodenal ligament. In addition, patients must be in an acceptable condition to undergo major surgery (encompassing extended hemihepatectomy in most cases). Locally advanced disease is based on the extent of proximal infiltration into the biliary ductal tree, the portal venous system, and the hepatic artery or its branches. Therefore, unresectability of patients with HCCA can result from local (including vascular) and nodal status, as well as the presence of distant metastases and comorbidity of the patient. Although ill-defined, these different causes of unresectability are likely to influence survival. The aim of this study was to evaluate overall survival in a group of patients with unresectable HCCA and to define prognostic factors in these patients.

## Materials and Methods

### Patients and staging

All consecutive patients suspected of HCCA who presented in the AMC over a 7-year period (from March 2003 to March 2009) were evaluated. Only patients with a tumor arising from the biliary confluence or the right or left main hepatic ducts were included. Patients with tumors originating in the proximal common hepatic duct were included if the tumor extended into the biliary confluence (Bismuth–Corlette classification II–IV). Cross-sectional imaging studies such as CT and MRI were used in addition to ultrasound with Duplex to assess liver parenchymal invasion, vascular invasion in the portal venous system and/or hepatic arteries, and hepatic metastases, lymph node metastases, and extrahepatic metastases, as previously described [10].

After imaging studies were concluded, resectability was discussed in a hepatobiliary multidisciplinary meeting. Patients considered to have potentially resectable tumors underwent further evaluation with a staging laparoscopy, and in the last 2 years of this study, with an additional PET-CT [11]. Staging laparoscopy was routinely performed in resectable patients when feasible, although staging laparoscopy was omitted in some patients with limited BC type I or II tumors, as we recently described in more detail [12]. Furthermore, ^99m^Tc-mebrofenin scintigraphy [13] was performed in conjunction with CT volumetry to determine the function of the future remnant liver [13]. Portal vein embolization was performed when the future remnant liver function or volume (>35–40 %) was deemed insufficient [14]. When no distant metastases were found during further evaluation, patients were planned for resection, and preoperative biliary drainage was performed on at least the future remnant liver, either percutaneously or endoscopically [15].

### Treatment

During laparotomy, the abdomen was inspected for peritoneal seeding or liver metastases, and for lymph node involvement outside the liver hilum, in the hepatoduodenal ligament, and along the common hepatic artery until the celiac axis. In patients deemed to be resectable, a radical resection was undertaken encompassing excision of the liver hilum en bloc with (extended) hemihepatectomy, including the caudate lobe, and complete lymphadenectomy of the hepatoduodenal ligament [2, 10, 16]. The portal vein bifurcation was excised and reconstructed when involved by tumor.

In case of unresectability, only a cholecystectomy was performed. In some selected cases diagnosed as Bismuth type I or II tumors with incurability due to locally advanced disease, a palliative (R1-2) local hilar resection was performed with biliary-enteric anastomoses. These patients with incurable disease were scored as unresectable. In patients with unresectable disease, biliary drainage was accomplished by definitive internal stenting by either PTC or ERCP using metal expandable stents, usually within the same hospital admission. Adjuvant therapy was not administered after resection in the patients of this study period. The use of palliative therapy was not protocol driven and was administered at the discretion of the surgeons and medical oncologists. Recently, palliative treatment consisting of gemcitabine plus cisplatin is routinely advised for unresectable HCCA patients in view of the encouraging results of the ABC-02-trial [17].

Brush cytology obtained during ERCP has low sensitivity and often does not result in a definitive tissue diagnosis; thus, pathological proof of malignancy is hard to obtain in all HCCA patients [18, 19]. As a result, and since pathological proof was not essential in patients undergoing palliative stenting only, cytological or histological proof was not available in all patients (Table 1).

**Table 1:** Characteristics of 217 HCCA patients seen from March 2003 through march 2009

Characteristics	Patients (%)
Male	139 (64)
Female	78 (36)
Median age (range)	64 (32–88)
Bismuth type	
Type I or II	31 (14)
Type IIIa	77 (36)
Type IIIb	42 (19)
Type IV	67 (30)
Medical history	
Other malignancy	21 (10)
Cholelithiasis/cholecystectomy	27 (12)
PSC	7 (3)
Hypercholesterolemia	14 (6)
Diabetes mellitus type II	21 (10)
Choledochal cyst	4 (2)
Resectability	
Unresectable at imaging or staging laparoscopy	122 (56)
Unresectable at laparotomy	38 (17)
Resectable	57 (26)
Histology	
Malignant	164 (75)
Benign	4 (2)
Unknown	49 (23)
Died during follow-up	42 (19)
Alive during follow-up	7 (3)
(Palliative) treatment	
Chemotherapy	22 (10)
Photodynamic therapy	8 (4)
Radiotherapy	8 (4)
Biliary drainage	210 (96)

### Scoring

The reason for unresectability was scored at the moment of the final assessment (during the hepatobiliary meeting, after staging laparoscopy, or during exploration). Thus, when a patient was considered unresectable following imaging based on locally advanced disease, the patient was scored as locally advanced, even when liver metastases appeared later in the course of the disease. Likewise, a patient who was diagnosed with peritoneal metastases during exploration was scored as extrahepatic metastases. If more causes of unresectability were found, the cause of unresectability was scored in the following decreasing order: liver metastases, extrahepatic metastases, positive lymph nodes, locally advanced disease. Patients who refused resection or were not in a condition to undergo major surgery were not included in the survival analysis.

Almost all patients initially underwent biliary drainage procedures using plastic stents inserted by either ERCP or PTC. After confirmation of unresectable disease, plastic stents were generally replaced by metal stents. When patients received a metal stent after initial plastic stenting, this was scored as metal stent.

### Survival

Survival data were obtained from our database or that of local hospitals and, if necessary, updated by contacting the primary-care physicians. Furthermore, additional survival data were collected through contacting registry databases. Survival (in months) was measured from the date of initial presentation at our center to the date of death or the date of last contact when alive.

### Statistical analysis

The data were analyzed using SPSS ver. 16.0 software (SPSS, Inc., Chicago, IL, USA). Overall survival times were evaluated from the time of initial presentation at our center to the time of death. Kaplan–Meier estimates of survival were obtained. Prognostic factors for overall survival were evaluated using log-rank test statistics, and when significant in univariate analysis (*p* < 0.05), factors were also examined using multivariable Cox proportional hazards modeling. The following predictors were considered: age, sex, Bismuth stage, histological confirmation, reason of unresectability, and palliative treatment. *P* values < 0.05 were considered statistically significant.

## Results

### Patient characteristics and resectability

The clinical characteristics of the study population are summarized in Table 1. A total of 217 patients were included in this evaluation. Of these, 103 patients were deemed unresectable on the basis of imaging studies (Table 1). Another 18 patients were found to be unresectable during staging laparoscopy, mostly because of peritoneal or liver metastases. During exploration, a further 38 patients were judged to be unresectable due to peritoneal metastases (*n* = 5), liver metastases (*n* = 3), lymph nodes beyond the hepatoduodenal ligament (*n* = 13), or locally advanced disease (*n* = 14). Two of these patients underwent local hilar resection and biliary-enteric anastomosis. The remaining 57 patients (26 %) underwent resection. Surgical and pathological details of these patients are given in Table 2. The reasons for unresectability in all patients not resected (*n* = 160) were locally advanced disease (*n* = 81), extrahepatic metastasis (*n* = 20), liver metastasis (*n* = 16), lymph node involvement beyond the hepatoduodenal ligament (*n* = 23), and unfitness for major surgery (*n* = 19). Palliative chemotherapy, radiotherapy, or photodynamic therapy was administered in 22, 8, and 8 patients, respectively.

**Table 2:** Operation and pathological details of 57 resected HCCA patients

	Patients [*n* = 57 (%)]
Operations	
Hilar resection only	7 (12)
Hilar resection + left hepatectomy	17 (30)
Hilar resection + left extended hepatectomy	6 (10)
Hilar resection + right hepatectomy	8 (14)
Hilar resection + right extended hepatectomy	17 (30)
Central resection	2 (4)
Combined with Whipple resection	2 (4)
Resection margins	
Radical resection (R0)	43 (75)
Nonradical resection (R1)	9 (16)
Benign lesion	5 (9)
Pathological features of malignant lesions	*n* = 52
T-stage (according to 7th edition of TNM)	
T1 (confined to the bile duct)	11 (22)
T2a (invades beyond the wall of the bile duct)	15 (29)
T2b (invades adjacent hepatic parenchyma)	14 (28)
T3 (invades unilateral branches of the portal vein or hepatic artery)	7 (14)
T4 (invades main portal vein or its branches bilaterally; or the common hepatic artery)	5 (10)
Tumor predominantly papillary	10 (20)
Vascular invasion	12 (24)
Perineural invasion	38 (73)
Positive lymph nodes (N1)	12 (24)

Histological proof of malignancy was available in 168 of the 217 (77 %) patients. In four patients, final histology of the resection specimen showed benign inflammatory disease. No histological proof of malignancy was available in the remaining 49 patients, generally after several negative ERCP brush cytologies. Nonetheless, of these 49, 42 died during follow-up, making malignancy the likely cause.

### Survival

**Fig. 1:**
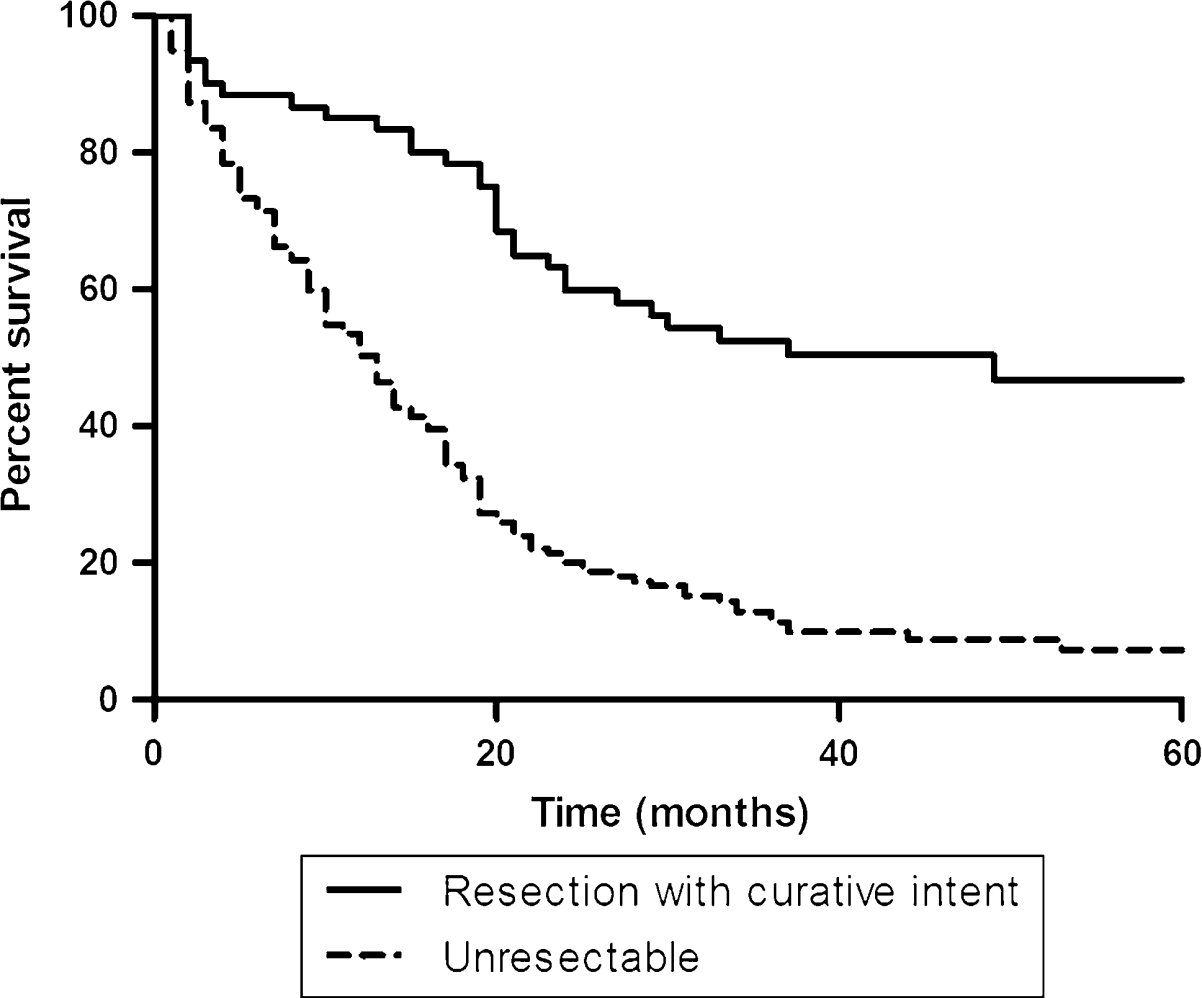
Survival of unresectable and resected patients with HCCA (*p* < 0.001)

Mean and median follow-up time of all patients were 21 and 16 months (range = 0–100 months), respectively, and for patients who had undergone resection, median survival was 30 months. Mean and median follow-up time of patients alive were 48 and 46 months (range = 16–87 months), respectively. Overall median and 1-, 3-, and 5-year survival rates were 33 months and 85, 49 and 42 %, respectively, for patients who underwent resection, excluding patients with benign disease. Including the patients with benign disease, these values were 37 months and 85, 51, and 43 %, respectively. Forty-four patients (83 % of resected patients with malignant pathology) had a R0 resection with a median survival of 49 months compared to a median survival of 19 months for patients with a R1 resection (*p* = 0.02). Overall median and 1-, 3-, and 5-year survival rates of unresectable patients were 13 months and 52, 12 and 7 %, respectively (Fig. 1). Survival of patients who underwent R1 resection was not significantly better than survival of unresectable patients (*p* = 0.22).

### Survival of unresectable patients

**Fig. 2:**
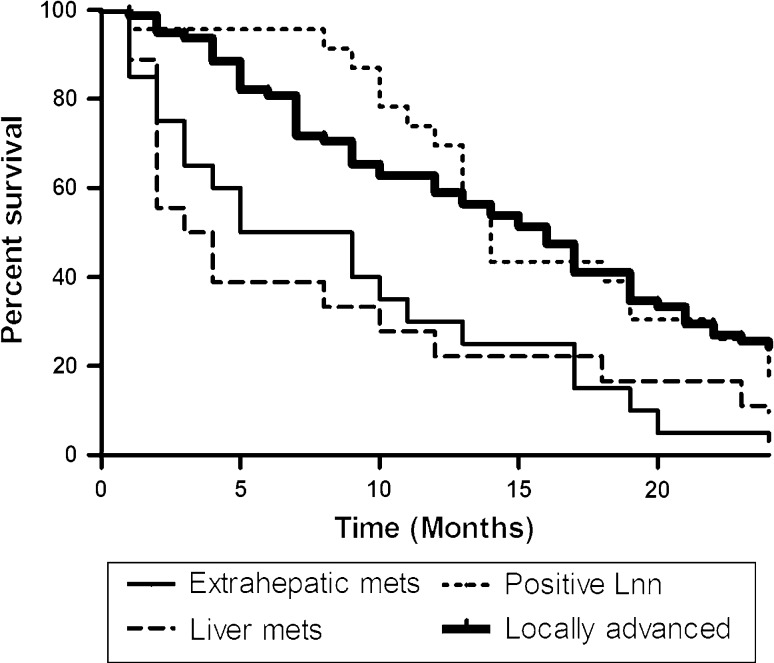
Survival of unresectable HCCA patients, stratified according to cause of unresectability

Univariate and multivariate analyses of clinicopathological parameters revealed that the cause of unresectability and biliary drainage with a metal stent were the only independent predictors of survival (Table 3). Patients with liver or extrahepatic metastases had a worse prognosis than patients with other causes of unresectability (Fig. 2). There was no difference in median survival in patients with (13 months) or without (10 months) histological confirmation of malignancy (*p* = 0.10).

**Table 3:** Uni- and multivariable analysis of predictive factors for survival in unresectable patients with HCCA

	Median survival (months)	*p*	Multivariable HR (95 % CI)	*p*
Age				
<64 years	13	NS	–	
≥64 years	12			
Gender				
Male	10	NS	–	
Female	14			
Histological confirmation				
Yes	13	NS	–	
No	10			
Bismuth type				
Type I or II	7	NS	–	
Type III	14			
Type IV	10			
Cause of unresectability		<0.01		<0.01
Locally unresectable	16		Reference	
Liver metastasis	3		2.26 (1.35–3.79)	<0.01
Extrahepatic metastasis	5		2.45 (1.39–4.33)	<0.01
Lymph node metastasis (N2)	14		0.96 (0.59–1.56)	NS
Therapy				
Laparotomy performed				
Yes	14	0.15	–	
No	10			
Chemotherapy				
Yes	13	NS	–	
No	12			
Biliary drainage				
Plastic stent	10	<0.01	0.66 (0.44–0.97)	0.03
Metal stent	14

Seventeen unresectable patients (12 %) survived longer than 36 months (range = 36–77 months); details of these patients are given in Table 4. Most patients received a metal stent, and when no metal stent was placed, the plastic stents were replaced usually every 3 months. Interestingly, six of eight patients still alive had no pathological confirmation of malignancy.

**Table 4:** Unresectable HCCA patients with long-term (>36 months) survival

Age	Sex	Bismuth type	Cause unresectable	Treatment	PA	Status
67	M	IIIb	Locally advanced	Wallstent	Unknown	Alive, 77 months
40	M	IV	Locally advanced	Wallstent	Malignant	Alive, 72 months
74	M	IV	Locally advanced	Wallstent	Unknown	Alive, 70 months
72	F	IIIa	Lymph node metastasis	Wallstent	Malignant	Died, 64 months
55	M	IV	Locally advanced	TACE, multiple plastic stents	Malignant	Alive, 60 months
52	M	IIIb	Locally advanced	Wallstent	Malignant	Died, 53 months
88	M	IIIa	Comorbidity	Plastic stent	Unknown	Alive, 50 months
46	M	IIIa	Patient refused resection	Experimental therapy, multiple plastic stents	Unknown	Alive, 49 months
75	F	IV	Locally advanced	Multiple plastic stents	Malignant	Died, 44 months
56	F	IIIa	Locally advanced	Multiple plastic stents	Malignant	Died, 45 months
75	M	IIIa	Patient refused resection	Multiple plastic stents	Unknown	Alive, 39 months
55	F	IIIb	Locally advanced	Multiple plastic stents	Malignant	Died, 39 months
64	M	IV	Lymph node metastasis	Multiple plastic stents	Unknown	Alive, 38 months
65	F	IV	Locally advanced	Wallstent	Malignant	Died, 37 months
60	M	IIIa	Lymph node metastasis	Wallstent, RTx	Malignant	Died, 37 months
77	M	IIIa	Co-morbidity	Wallstent	Unknown	Died, 36 months
72	M	II	Locally advanced	Palliative resection, RTx	Malignant	Died, 36 months

## Discussion

This study assessed survival in the denominator of HCCA patients referred to a tertiary referral center during a 7-year period and evaluated prognostic factors of unresectable patients. Twenty-six percent of these patients underwent resection. Five-year overall survival rates of patients who underwent resection and of unresectable patients were 43 and 7 %, respectively. This study represents one of the largest published series of patients with HCCA (more than 200 patients) that also included unresectable patients. The resectability rates vary substantially among published series (from 19 to 56 %) but are comparable with the 27 % found in the current series, as seen in Table 5. The variation in resectability rates inherently depends on the referral pattern of referring centers.

**Table 5:** Studies presenting resectability rates and survival data of patients with resectable and unresectable HCCA

Author	Resected patients / total patients	Survival resected patients	Median survival unresectable patients
Nakeeb [22]	17/72 = 24 %	5-year 34 %	NA
Kawasaki [7]	79/140 = 56 %	5-year 38 %^a^	10 months
Jarnagin [6]	106/279 = 38 %	Median 39 months^b^	11 months
Witzigman [8]	60/184 = 33 %	5-year 22 %	6 months
Connor [4]	55/288 = 19 %	NA	145 days
Ito [5]	38/91 = 42 %	5-year 31 %^b^	4 months^b^
Current series	57/217 = 26 %	5-year 43 %	13 months

*NA* not available

^a^Survival of patients with R0 resections

^b^Disease-specific survival

The 5-year-survival rate of 43 % in patients who had undergone resection is high in comparison with other series, as seen in Table 5, but is consistent with survival data from a more recent series from Japan [16]. We have previously shown that long-term survival has steadily improved in specialized centers over the years worldwide [10], and the survival rate found in the current series seems a promising continuation of this upward trend.

Still, 74 % of HCCA patients in the current study were ultimately deemed unresectable and have a considerably worse prognosis with a median survival of 13 months and 5-year survival of only around 7 %. On the other hand, the 7 % long-term survival in unresectable patients with HCCA is rather surprising. We and others have shown that around 10–15 % of pathology specimens of patients suspected for HCCA ultimately show benign disease [20]. Furthermore, the diagnosis of malignancy can be false-positive as an effect of inflammation and stents. The diagnosis of adenocarcinoma was confirmed histologically in 77 % of patients, which is higher or in accordance with other studies [8, 21]. However, a few patients in the group with histologically unconfirmed malignancy may have harbored benign disease, which in part explains the prolonged survival of unresectable patients found in this study. However, there was no difference in survival of patients with confirmed or unconfirmed unresectable disease, whereas most patients with unconfirmed disease died during follow-up (42 of 49), suggesting a malignant cause of their hilar obstruction. Lastly, long-term survival was also found in patients with histologically confirmed malignancy, as seen in Table 4. Hence, besides patients with benign lesions mimicking malignancy, the majority of unresectable patients showing long-term survival apparently had indolent, slow-growing tumors, with a relatively good survival, provided that adequate biliary drainage was maintained to prevent death from unresolvable cholangitis. In our perception, many patients die not as a result of the expanding tumor but as a result of unresolvable cholangitis complicating the clinical course. Nonetheless, we have currently no data to support this and consequently this remains speculation.

We also looked at the impact on survival of metal stents versus plastic stents. This analysis was, however, substantially biased since almost all patients initially received a plastic stent, which ideally was replaced by a metal stent when unresectability was confirmed. Since most patients who received a metal stent already had a plastic stent in place, a valid comparison could not be made.

Clearly, the most important prognostic factor for patients with HCCA is whether a resection can be performed. However, the only significant prognostic factor in multivariate analysis for patients with unresectable disease was the cause of unresectability; patients diagnosed with liver metastasis or extrahepatic metastasis fared significantly worse than patients with other causes of unresectability. This finding is not surprising, yet in discussing prognosis with a patient, it is helpful to present these median survival times associated with the different causes of unresectability. Furthermore, future studies exploring interventions in patients with unresectable HCCA should stratify the cause of unresectability since the cause substantially influences expected survival.

We realize the limitations of this study. First, data were retrieved in a retrospective manner with its inherent methodological drawbacks. Second, patients were treated in a single tertiary referral center and results obviously could be different in other hospitals. Finally, as described above, pathological confirmation of unresectability was not available in 25 % of patients and therefore maybe patients with benign disease mimicking malignancy were included. However, considering the median survival of only 10 months in patients without histological proof and the high number of these patients who died during follow-up, this group should be limited. Nonetheless, one should keep in mind that patients diagnosed with unresectable HCCA may ultimately have benign disease. Until we find more accurate techniques to differentiate benign from malignant biliary lesions, these patients will remain being diagnosed with HCCA in the future.

Notwithstanding the above-mentioned limitations, HCCA is a rare disease and because of the small numbers encountered, even in specialized centers, randomized studies are difficult to perform. As a result of the small numbers, the study period of many studies is quite long, introducing possible biases due to changes in management. By including only patients over a 6-year period, we minimized this effect. By assessing an unselected group of patients, we believe this study shows the daily management of this disease.

In conclusion, of the patients who presented with HCCA in our center, 26 % proved resectable. Five-year survival of resected and unresectable patients was 43 and 7 %, respectively, the latter including long-term survivors. The 5-year survival rate of 7 % for unresectable patients is remarkable in the absence of effective chemotherapy or radiation therapy, emphasizing the indolent growth of some of these tumors. Patients with metastases had a much worse prognosis with a median of 4 months.
